# Clinical efficacy evaluation of a traditional Miao technique of crossbow needle therapy in the treatment of knee osteoarthritis: a multi-center randomized controlled trial

**DOI:** 10.1186/s13063-020-04508-7

**Published:** 2020-06-22

**Authors:** Jing Fu, Hong-Cai Shang, Li-Ying Wang, Chen Zhao, Xiao-Fang Yang, Wei-Wei Guo, Chun-Xia Lu, Jin Cui, Yan-Ping Wang

**Affiliations:** 1 Guizhou University of Traditional Chinese Medicine, Guiyang, 550000 China; 2 Key Laboratory of Chinese Internal Medicine of Ministry of Education and Beijing, Beijing University of Chinese Medicine Affiliated Dongzhimen Hospital, Beijing, 100700 China; 3 Institute of Basic Research in Clinical Medicine, China Academy of Chinese Medical Sciences, Beijing, 100700 China; 4 No.2 Affiliated Hospital of Guizhou University of Traditional Chinese Medicine, Guiyang, 550000 China; 5 Chinese Medicine Hospital of Qiandongnan Miao and Dong Autonomous Prefecture, Kaili, 556000 China; 6 No.1 Affiliated Hospital of Guizhou University of Traditional Chinese Medicine, Guiyang, 550000 China

**Keywords:** KOA, Crossbow needle therapy, Acupuncture, Randomized controlled trial

## Abstract

**Background:**

Knee osteoarthritis (KOA) seriously reduces quality of life and is a major threat to the health of the middle-aged and elderly. This study aimed to assess the efficacy of Miao crossbow needle therapy vs. acupuncture for KOA therapy.

**Methods:**

This multicenter, randomized controlled trial was performed at three hospitals between April 2016 and December 2016. The patients were randomized to receive crossbow needle (CN) or acupuncture (AT). All treatments were completed within 46 days. Evaluation of treatment was conducted on the 46th, 62nd, and 77th days. The primary endpoint was change of Western Ontario and McMaster Osteoarthritis Index (WOMAC) score on the 46th day. The secondary endpoints included WOMAC score, the Lysholm knee score, the Japanese Orthopedic Association (JOA) knee score, visual analog scale (VAS), and the MOS 36-item short-form health survey (SF-36), on the 46th, 62nd, and 77th day.

**Results:**

Finally, data of 301 participants were analyzed for the efficacy of treatment. Compared with AT, there was a larger change of WOMAC score in the CN group after treatment [− 25.0 (95% CI − 27.0, − 23.0) vs. − 18.8 (95% CI − 20.8, − 16.9), *P* < 0.001]. In the CN group, the WOMAC score was lower at all three time points (*P* = 0.008, *P* = 0.003, *P* < 0.001 respectively), while the Lysholm knee score (*P* = 0.03) and JOA score (*P* = 0.013) were higher and the VAS score (*P* = 0.011) was lower on the 77th day.

**Conclusion:**

Both Miao crossbow needle therapy and acupuncture reduced the WOMAC score. Miao crossbow needle therapy can be an alternative method for treating patients with knee osteoarthritis.

**Trial registration:**

ChiCTR, ChiCTR-INR-16008032. Registered on 12 March 2016.

## Background

Knee osteoarthritis (KOA) is a common chronic progressive disease, mainly involving articular cartilage retrogression and secondary hyperostosis. Its onset is associated with multiple factors like age, body mass index (BMI), sex, inflammation, long-term irrational exercise, and genetics. The main clinical manifestations include joint swelling, stiffness, and dysfunction. KOA and obesity are considered to be the most common chronic diseases affecting the health of Americans aged 50 to 84 [1]. More than half of the population under 65 years of age suffer from symptomatic KOA with symptoms persisting for more than 3 years [2]. In China, although the prevalence varies among cities and areas, it is increasing year by year; a conservative estimate shows that about 200,000 Chinese patients undergo joint replacement surgery each year [3]. Since the essence of KOA is the degenerative changes of the articular cartilage, the prevalence will surely increase with the aging of the population. Hence, this disease has become a major threat to the health of middle-aged and elderly people.

The repair capacity of articular cartilage appears to be extremely limited, and damage is often irreversible. All existing medical measures (drugs, surgery, or physical treatment) fail to reverse the pathological changes of damaged articular cartilage. Therefore, the treatment of KOA to a great extent aims to relieve or eliminate pain, reduce inflammation, improve or restore joint function, delay disease progression, and improve patients’ quality of life [4]. To this end, Western medicine mainly uses drugs and surgery. Along with quick effects, drugs may have some toxic and side effects, and their long-term use may increase the risk of suffering from adverse gastrointestinal reactions. Surgical treatment has multiple contraindications and complications and is expensive.

Knee pain belongs to the category of chronic pain of the motor system. As early as 1996, during a conference in Milan (Italy), it was recognized as one of the indications for acupuncture [5]. Furthermore, acupuncture, as physiotherapy, is relatively safe. A large number of clinical studies have confirmed that this therapy can significantly relieve pain and improve joint function in patients with KOA. It has been recognized by scholars in China and in the world and accepted by patients as a complementary therapy to KOA.

Among more than 40 kinds of external treatment methods, the crossbow needle therapy used by the Miao ethnic minority in China is a popular and broad-spectrum treatment method that integrates the effects of acupuncture and drugs. Miao medicine is commonly adopted to manage conditions like rheumatism, “*leng gu feng*”, and “*mao tou feng*” [6–9], while in modern clinical studies, it is mainly used to treat KOA. Nevertheless, all of the existing studies conducted previously [10–13] were single-center and small-sized clinical studies, resulting in inadequate clinical evidence. Therefore, they are insufficient to accurately and fairly evaluate the clinical efficacy as well as the characteristics of crossbow needle therapy of the Miao ethnic minority in the treatment of KOA.

In this study, a multi-center, randomized, non-inferiority trial was used to determine whether the clinical efficacy of crossbow acupuncture used in Miao medicine was not inferior to that of acupuncture, so as to provide a feasible alternative to acupuncture for patients with knee osteoarthritis. The protocol for this trial has been published [14].

## Methods

### Study design and setting

This was a parallel, non-inferiority, randomized controlled clinical trial aiming to compare the effect of crossbow needle therapy vs. regular acupuncture therapy in patients with KOA [14] The patients were enrolled among the outpatients and inpatients from the acupuncture departments of the No.1 Affiliated Hospital of Guizhou University of Traditional Chinese Medicine, No.2 Affiliated Hospital of Guizhou University of Traditional Chinese Medicine, and Chinese Medicine Hospital of Qiandongnan Miao and Dong Autonomous Prefecture from April 13, 2016, to December 29, 2016. The eligible patients were randomly assigned to the crossbow needle group and the acupuncture group in a 1:1 ratio [14].

### Study population

#### Diagnostic criteria

The diagnostic criteria [15] for KOA are:

1. Frequent knee pain in the past 1 month;
2. Osteophytes at the joint margin by X-ray;
3. Diagnosis confirmed by joint fluid examination;
4. Aged > 40 years;
5. Morning stiffness of < 30 min;
6. Bony crepitus.

Those who meet criteria 1 and 2, or 1, 3, 5, and 6, or 1, 4, 5, and 6 are usually diagnosed with KOA.

#### Inclusion criteria

Patients who met all of the following criteria were enrolled in the trial:

1. Aged between 40 and 75 years;
2. Meeting the above diagnostic criteria 1 and 2 (patients graded 1, 2, or 3 osteoarthritis by Kellgren-Lawrence scale [16]) or 1, 4, 5, and 6;
3. No intervention performed within 1 week before enrollment;
4. Well aware of all the tasks involved in the trial and prepared to comply with treatment;
5. Willingness to sign the informed consent form.

#### Exclusion criteria

Patients who met any of the following criteria were excluded from the trial:

1. Pregnant or lactating women;
2. Susceptible to allergy;
3. Other knee disorders;
4. Contusion or sprain in ankle or foot, or other disorders that affect normal walking;
5. Ankle/foot deformity or pain;
6. Skin disorders or swelling at the treated site;
7. Combined with other diseases that require drugs that will have a therapeutic effect on knee osteoarthritis, such as protrusion of lumbar intervertebral disc, cervical spondylosis, scapulohumeral periarthritis, and migraine which using analgesic drugs for treatment.

### Sample size

The trial was designed to determine the efficacy of the crossbow needle therapy on KOA and show that its clinical efficacy is not inferior to acupuncture therapy. Therefore, we chose a non-inferiority trial design [17]. Sample size calculation was based on the formula $n=2{\left[\frac{\left(\mu \alpha \kern0.5em +\kern0.5em \mu \beta \right)s}{\delta}\right]}^2$, in which *μα* and *μβ* are constants and *s* is the combined standard deviation. We set *α* at 0.05, *β* at 0.10, *δ* at 1.5, and *s* at 4.0 (*δ and s* were based on expert consensus to determine the formula parameters), with *μα = μ*_0.05_ = 1.645 and *μβ = μ*_0.10_ = 1.282. After calculation, the sample size of each group was around 122. Assuming a 20% dropout rate, each group needed at least 153 patients, and so 306 patients were recruited. In this trial, only one knee was included for each patient. For patients with bilateral KOA, the most affected side was evaluated.

### Randomization and blinding

Stratified block randomization was carried out by third-party statisticians who were not involved in any other part of the trial. Central randomization was conducted by CLINDA Soft Co., Ltd. (Tianjin, China). A random sequence was generated using SAS 9.2 (SAS Institute, Inc., Cary, NC, USA) by an independent statistician who was not involved in the study, and the sequence was stored in a central randomization system (interactive web response (IWR) system). The patients were stratified based on the three different centers and randomly assigned to the crossbow needle group (CN group) and the acupuncture group (AT group) in a 1:1 ratio. The crossbow needle group received crossbow needle therapy. Regular acupuncture therapy was performed on the acupuncture group.

In this trial, patients and therapists could not be blinded due to the fact that the crossbow needle treatment area was large and it was very different in shape from the needles used in acupuncture. However, the data collection research personnel and data analysts were blinded to the patient grouping. And the data collection research personnel enrolled the participants; the independent statistician who was not involved in the other part of study assigned the participants.

### Interventions

All treatments were conducted in the outpatient treatment rooms of the three centers. The therapists were not involved in the other parts of the trial. The treatments were performed three times per week, for a total of 20 treatments. All the treatments were completed within 46 days. The assessments of the treatments were performed after treatment, on the 46th day, the 62nd day, and the 77th day. All practitioners involved in the trial were trained and registered professionals. Acupuncture was performed by primary physicians with > 5 years of experience at each center. A training program to standardize all manipulations and filling of a case report form (CRF) was provided before the trial. The investigators informed all patients about the possible inconveniences from the trial. In this trial, since only one side was included, only the included side was treated during the trial (for the knee joint not included in the trial, we provided the same 20 treatments free of charge to the patients who needed them at the end of the trial).

### Crossbow needle group

**Fig. 1:**
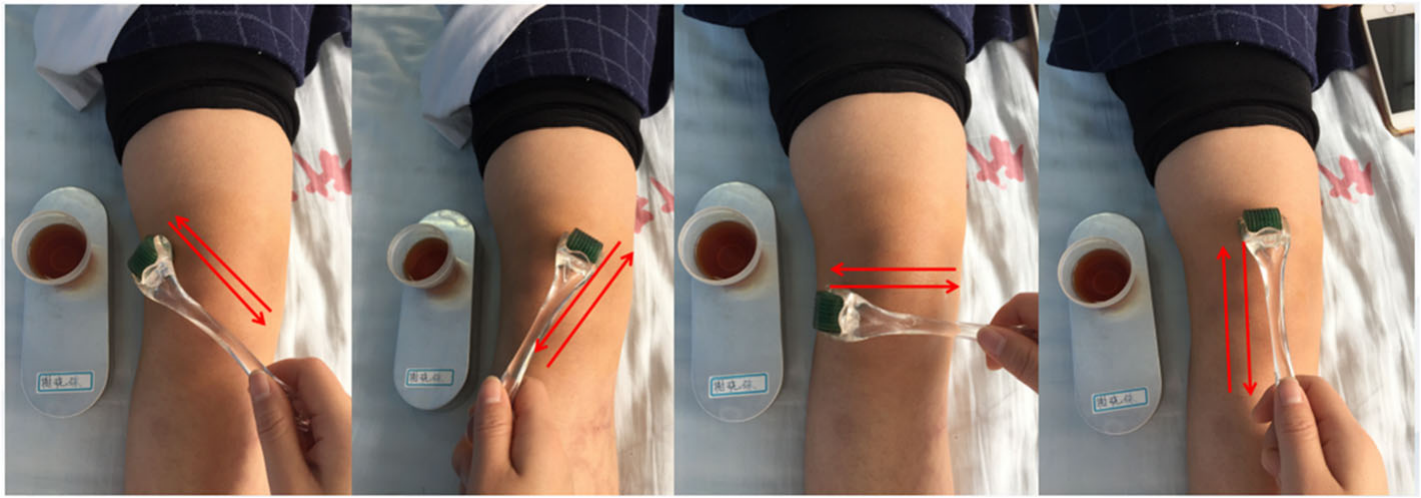
Crossbow needle treatment. The red arrows indicate the rolling directions of the skin needle. The container beside the leg contains the herbal liquid

Before treatment, a prescription of herbs containing Sheng Cao Wu (*Radix Aconiti Kusnezoffii*) (20 g), Tou Gu Xiang (*Gaultheria yunnanensis*) (50 g), Hei Gu Teng (*Periploca forrestii*) (30 g), and Ba Jiao Feng (*Alangium chinense*) (15 g) was ground into a powder, put into a glass container, and soaked in 1000 ml of 50-proof white liquor for 7 days. The obtained clear brown liquid is effective in dispelling wind and eliminating dampness, dispersing numbness and relieving pain, dredging collaterals, and promoting blood circulation [10–13]. The patients in this group received crossbow needle therapy using 0.22 × 1.0 mm skin needles (Cloud & Dragon Medical Device Co., Ltd., Wujiang, Zhejiang, China). The practitioner soaked the skin needles in the brown herbal liquid for 10 min before treatment. The patients were asked to take a supine or sitting position. The soaked skin needles were applied to the square knee area formed by Xuehai (SP10), Liangqiu (ST34), Yanglingquan (GB34), and Yinlingquan (SP9) [6], with the patella as the center, moving in different directions (Fig. 1). One application involved rolling the skin needle in all directions, as displayed in Fig. 1. The skin needles were dipped into 5 ml of the herbal liquid after every five applications until the liquid was used up. The intensity of application varied from person to person, depending on the tolerance of the patients.

### Acupuncture group

The acupuncture group was treated with 0.30 × 40 mm, and 0.30 × 50 mm disposable sterilized filiform needles (Suzhou Medical Appliance Co., Ltd., Suzhou, Jiangsu, China) applied to the Dubi (ST35), Neixiyan (EX-LE4), Xuehai (SP10), Liangqiu (ST34), Yanglingquan (GB34), and Ashi points. The selection of the points was based on the basic theory of acupuncture therapy [18, 19] to treat KOA. Practitioners performed the treatment according to the national standard of acupuncture manipulation [20, 21]. The needles were inserted and manually manipulated until de qi was achieved.

### Combined use of drugs

During the whole period of observation, it was suggested that the patients should not use drugs or other methods for the treatment of KOA in addition to the experimental scheme. During the treatment period, if the patients took medication by themselves or used other methods of treatment due to aggravated symptoms and unbearable knee pain, they were withdrawn from the trial. The clinicians provided appropriate treatments according to the specific conditions of the patients until the symptoms improved.

### Endpoint measures

#### Primary endpoint measure

The Western Ontario and McMaster Universities Osteoarthritis Index (WOMAC) was used to measure the pain and function before and after the therapy [22]. The specific primary endpoint was change in WOMAC score from baseline to the 46th day when treatment finished. The WOMAC is a questionnaire composed of 24 questions. It was developed for patients with osteoarthritis of the knee and measures three subscales: pain, stiffness, and joint function. Higher scores indicate more severe symptoms.

#### Secondary endpoints measures

The secondary endpoints were WOMAC score, Lysholm knee score, Japanese Orthopedic Association (JOA) score, visual analog scale (VAS) score, and MOS 36-item short-form health survey (SF-36) at the three time points after finishing treatment.

The Lysholm knee score was used to assess the knee function of the patients. The score was rated from 0 to 100, with 25 points attributed to pain, 15 to locking, 10 to swelling, 25 to instability, 10 to stair climbing, and 5 each to squatting, limping, and support. Higher scores indicate milder symptoms.

The JOA score is a frequently used scale. It comprises ratings of pain (40 points), range of motion (20 points), gait (20 points), and activities of daily living (20 points). The JOA score was used to assess the therapeutic effect, with a higher score indicating a better effect.

The VAS score was applied to evaluate the intensity of pain experienced by the patients. It is a standard tool in pain studies to measure pain intensity. The score ranges from 0 to 100 (0 indicating no pain and 100 indicating the worst pain imaginable).

The SF-36 score was used to assess the patients’ living standards before and after treatment. The health survey consists of eight dimensions: physical function, role limitations due to physical problems, bodily pain, general health, vitality, social function, role limitations due to mental problems, and mental health. Higher scores represent a better quality of life.

### Statistical analysis

**Fig. 2:**
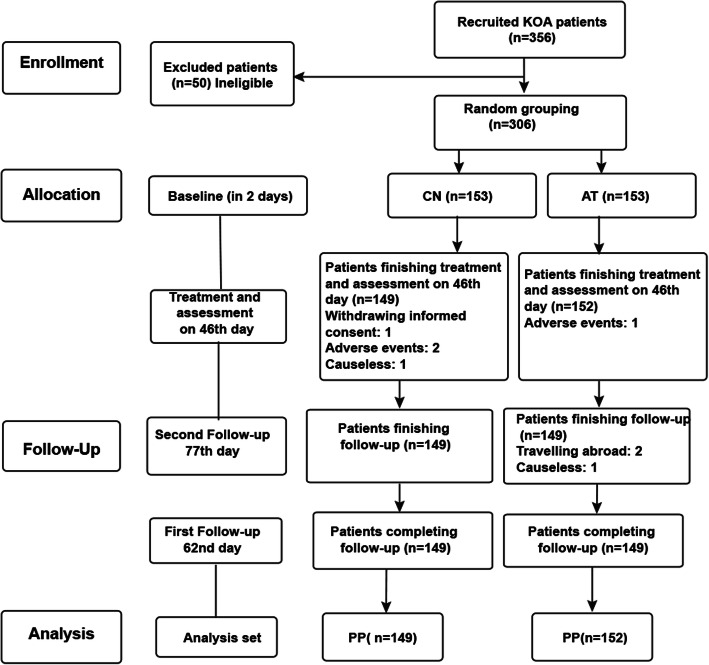
Participant flowchart. Baseline data were obtained within 2 days of the beginning of the trial. The treatment was completed on the 46th day; the first assessment of efficacy was performed on the 46th day. The first follow-up was on the 62nd day of the trial. The second follow-up was on the 77th day of the trial

Statistical analysis was conducted using SAS 9.2 (SAS Institute Inc., Cary, NC, USA) and SPSS 22.0 (IBM, Armonk, NY, USA). The graphical drawing software was GraphPad Prism 6.0. Data analysis was conducted in accordance with the per-protocol principle, where patients who were treated according to the trial protocol were included in the analysis (Fig. 2). The missing follow-up data were filled in by the carry-over of the previous measurement data. Shapiro-Wilk tests were used find whether the data conformed to normal distribution. Normally distributed continuous data are expressed as means ± SD and non-normally distributed continuous data are expressed as medians (Q1, Q3). Categorical data are expressed as *n* (%).

Analysis of covariance (ANCOVA) was used to correct the difference between baseline WOMAC score and the primary endpoint outcome after treatment. Meta-analysis and mixed model analysis were both used to examine the center effects that may have influenced the results because of the multi-center nature of the study.

Secondary endpoints were compared between groups by independent sample *T* test or Mann-Whitney *U* test. The chi-square test or Fisher’s exact probability test were used to compare qualitative indicators between groups. All tests were two-sided. A *P* value < 0.05 was considered statistically significant.

## Results

### Participants

From April 13, 2016, to December 29, 2016, a total of 356 patients were screened from the three centers; 50 ineligible patients were excluded, and 306 patients were enrolled. Five patients with missing primary endpoint outcome data were excluded. In the end, there were 149 patients in the CN group and 152 patients in the AT group (Fig. 2).

### Demographic and baseline data

There were no significant differences between the two groups regarding demographic data (age, sex, height, weight, BMI, and disease course), JOA score, Lysholm score, VAS score, and SF-36 score at baseline (all *P* > 0.05), except WOMAC score (*P* = 0.032) (Table 1).

**Table 1:** Comparison of the baseline data between the two groups

Measures	CN (*n* = 149)	AT (*n* = 152)	*P*
Sex, *N* (%)			0.279
Male	18 (12.1%)	25 (16.4%)	
Female	131 (87.9%)	127 (83.6%)	
Age, median (Q1,Q3), years	58 (51, 63)	60 (53, 65)	0.127
Height, median (Q1,Q3), cm	158 (154, 161)	157 (153.5, 161.5)	0.808
Weight, median (Q1,Q3), kg	60 (55, 65)	60 (54.3, 65)	0.773
BMI, median (Q1,Q3), kg/m^2^	24.4 (22.2, 26.1)	24.4 (21.9, 26.6)	0.954
Disease course, median (Q1,Q3), months	46 (24, 72)	48 (24, 84)	0.647
WOMAC score^a^, median (Q1,Q3)	34 (25, 45)	30 (18, 44)	**0.032**
JOA score^b^, median (Q1,Q3)	75 (70, 85)	75 (70, 85)	0.754
Lysholm knee score^b^, median (Q1,Q3)	59 (50, 66)	58 (47, 67.5)	0.935
VAS score^a^, mean (SD)	56.1 ± 16.6	55.9 ± 18.3	0.939
SF-36 score^b^, median (Q1,Q3)	455.5 (336.3, 569.7)	461.65 (369.6, 581.5)	0.358

*WOMAC score* Western Ontario and McMaster Osteoarthritis Index score, *JOA score* Japanese Orthopedic Association knee score, *VAS* visual analog scale, *SF-36* the MOS 36-item short-form health survey

^a^Disease severity increases as scores rise

^b^Disease severity decreases as scores rise

### Primary endpoint

In analysis of covariance after calibration of baseline WOMAC score, there was a statistically significant difference in the change of WOMAC score at the 46th day between the CN group [− 25.0 (95% CI − 27.0, − 23.0)] and the AT group [− 18.8 (95% CI − 20.8, − 16.9)] (*P* < 0.001). Mean difference (95% CI) was − 6.2 (− 8.9, − 3.4); compared with the non-inferiority bound value 1.5, the non-inferiority hypothesis was satisfied.

**Fig. 3:**
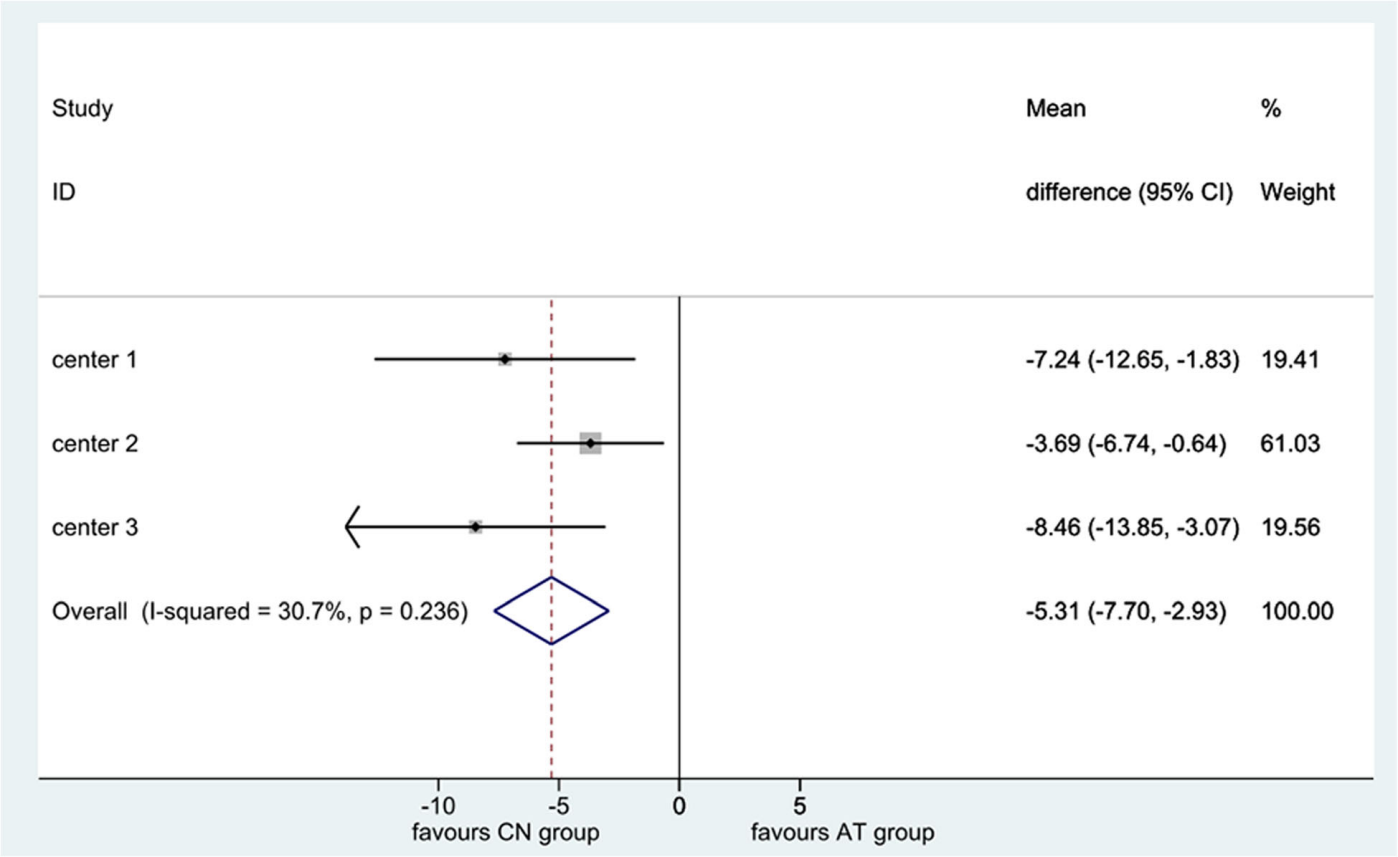
Test of the center effect. The heterogeneity among the three centers was low

Meta-analysis was used to test the center effect, and the heterogeneity among the three centers was low and the research conclusions were consistent as shown in Fig. 3. When mixed model analysis was used to test the center effect, this showed − 2 restricted log likelihood = 2380.692, intercept variance estimate = 55.742, *P* = 0.331, indicating that different centers had no significant hierarchical aggregation, that is, no central effect.

### Secondary endpoints

**Fig. 4:**
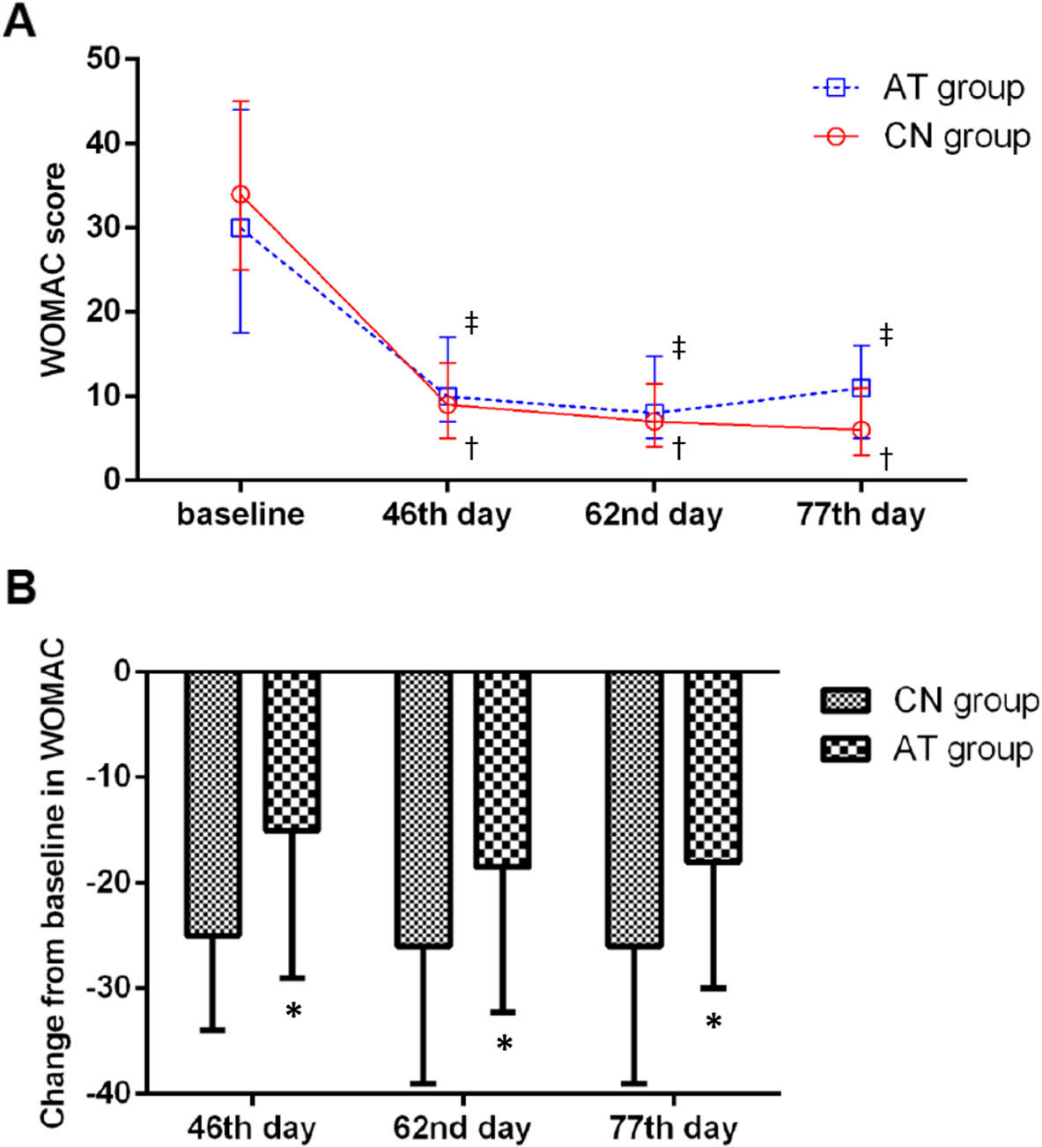
Assessment of WOMAC score. ‡, compare with baseline *P* < 0.05 in AT group; †, compare with baseline *P* < 0.05 in CN group; *, compare with CN group *P* < 0.05. **a** The WOMAC scores from baseline (assessment was made within 2 days before treatment started) to 77th day. The scores at three time points (46th, 62nd, and 77th day) were all significantly different (*P* < 0.05) between CN and AT groups. **b** The change in WOMAC score from baseline at three time points after treatment (All *P* < 0.05, between two groups)

Comparisons between the CN group and AT group showed the WOMAC score had statistically significant differences at all three time points; the Lysholm score, JOA score, and VAS score showed statistically significant differences on the 77th day. There was no significant difference in SF-36 score between the two groups (Table 2). The value of the WOMAC scores at each time point and the change in WOMAC score are also shown in Fig. 4.

**Table 2:** Comparison of secondary endpoints for the two groups at three time points after treatment

	CN (*n* = 149)	AT (*n* = 152)	*P*
**WOMAC score**^**a**^**,** median (Q1,Q3)			
46th day	9 (5, 14)	10 (7, 17)	0.008
62nd day	7 (4, 11)	8 (5, 14.5)	0.003
77th day	6 (3, 11)	11 (5, 16)	< 0.001
**Lysholm knee score**^**b**^**,** median (Q1,Q3)			
46th day	78 (69, 85)	76 (66, 85)	0.791
62nd day	81 (75, 90)	80 (70, 87)	0.101
77th day	81 (74, 93)	80 (70, 88)	0.030
**JOA score**^**b**^**,** median (Q1,Q3)			
46 days	90 (85, 95)	90 (80, 95)	0.648
62 days	95 (85, 100)	90 (80, 95)	0.157
77 days	95 (85, 100)	90 (80, 95)	0.013
**VAS score**^**a**^**,** median (Q1,Q3)			
46 days	30 (17, 40)	30 (20, 43)	0.115
62 days	21 (13, 35)	25 (16, 37)	0.162
77 days	20 (8, 33)	28 (10, 40)	0.011
**SF-36 score**^**b**^**,** median (Q1,Q3)			
46 days	536.7 (454, 636)	562.9 (464.6, 636)	0.816
62 days	599.3 (514.3, 664)	590 (482.9, 651.6)	0.294
77 days	612.5 (518.2, 674)	585.9 (487.3, 662)	0.179

*P* represents the comparison between the two groups at the corresponding time points

*WOMAC score* Western Ontario and McMaster Osteoarthritis Index score, *JOA score* Japanese Orthopedic Association knee score, *VAS* visual analog scale, *SF-36* the MOS 36-item short-form health survey

^a^Disease severity increases as scores rise

^b^Disease severity decreases as scores rise

### Safety

Sixteen patients (5.2%) reported adverse reactions in the two groups during the trial: six (3.9%) in the CN group and 10 (6.5%) in the AT group. The adverse reactions in the CN group were all redness and slight itching of the knee joint skin at the procedure site. The patients were advised not to scratch, and the symptoms disappeared the next day without treatment. In the AT group, adverse events included four (2.6%) cases of subcutaneous hematoma (all caused by patients’ massage upon the treatment site due to slight pinprick itching), three cases (2.0%) of bending of the needle (caused by patients’ voluntary changes of body position during acupuncture without doctor’s permission), two cases (1.3%) of sticking of needle (caused by hypertension as the patients received acupuncture for the first time), and one case (0.7%) of dizziness during acupuncture treatment (caused by undergoing treatment on an empty stomach, resulting in slight dizziness and sweating after needling) (Table 3). All adverse reactions were properly handled until symptoms disappeared. Two patients in the CN group were withdrawn from the trial due to adverse reactions, and one in the AT group was withdrawn from the trial due to adverse reactions.

**Table 3:** Adverse events in the two groups

	CN (*n* = 153)	AT (*n* = 153)	*P*
Red and itchy skin at the knee joint, *n* (%)	6 (3.9)	0	0.030
Subcutaneous hematoma, *n* (%)	0	4 (2.6)	0.123
Needle bent, *n* (%)	0	3 (2.0)	0.248
Sticking of needle, *n* (%)	0	2 (1.3)	0.498
Fainting during acupuncture treatment, *n* (%)	0	1 (0.7)	1.000

Assessed using intention-to-treat set

## Discussion

This study shows that the efficacy of Miao crossbow needle therapy is no worse than that of acupuncture therapy. The WOMAC score was lower after treatment in the CN group on the 46th, 62nd, and 77th day. In addition, the VAS score was lower and the Lysholm knee score and JOA score were higher in CN group. The adverse events of crossbow needle therapy and acupuncture therapy were not significantly different.

This study used acupuncture as the comparison. A randomized controlled trial in 570 patients with KOA during the course of 26 weeks concluded that acupuncture could provide significant improvement in joint function and pain relief compared with sham acupuncture for the treatment of KOA [23]. By carrying out a randomized controlled trial in 294 patients with KOA, Witt et al. [24] reported significant improvement in pain relief and joint function in the acupuncture group after 8 weeks of treatment and the efficacy of the acupuncture group was significantly better than that of the sham acupuncture group and the wait-and-see group. Du et al. [25] reported that acupuncture had been unanimously recognized to be effective for 110 diseases, and most frequently for KOA. These randomized controlled trials and review suggest that acupuncture has a positive clinical efficacy for KOA.

Chinese studies on acupuncture indicated that early KOA belongs to the grade II evidence-based acupuncture disease spectrum and patients may receive an overall improvement with acupuncture-based therapy or acupuncture therapy alone [26]. Advanced KOA belongs to the grade III evidence-based acupuncture disease spectrum and may be relieved through an acupuncture-based therapy, complementary acupuncture, or single acupuncture therapy [26]. In 2008, KOA was listed in the top 10 most frequent diseases initially diagnosed in the acupuncture clinic throughout three of six major administrative regions in China (Northeast, Eastern, and Southwest areas) [27]. The efficacy of acupuncture has been recognized by the majority of doctors and patients, and the treatment of KOA with acupuncture tends to be routine management. Many patients will involuntarily withdraw oral medications after undergoing acupuncture treatment due to, on one hand, the fear of toxic and side effects of drugs and, on the other hand, the recognition by Chinese patients for the efficacy of acupuncture. Therefore, taking acupuncture therapy as the control group contributes to the consistency of trial results with clinical practice.

A needle roller is usually used to replace the traditional crossbow medicinal needle in modern clinical practice [28], where the essence of its action mechanism is transdermal drug delivery. In both ancient documentary records and with modern clinical improvements, the basis of Miao crossbow needle therapy lies in multiple needles that shallow puncture the skin, which opens the stratum corneum to facilitate the absorption of topical drugs while discharging the body “toxins” (according to Miao medicine theory, most human diseases are caused by “toxins”), thereby achieving the purpose of treating local or systemic diseases [29]. The multiple microneedles arranged neatly on the roller pierce the cuticle of the skin, forming hundreds of microneedle pinholes; through these tiny pinholes, the drugs penetrate into the dermis of the skin, enter the capillary network, and reach the whole body via the blood. Transdermal administration not only increases the infiltration of the drugs to achieve the purpose of sufficient and rapid administration but also reduces the required dose of drugs and improves the efficacy of drugs [30, 31].

As a combination therapy, the efficacy of crossbow needle therapy might be associated with two factors: needles and drugs. First, the repeated mechanical stimulation of the needle roller on the skin surface can convert mechanical energy to thermal energy, which will dilate the small local vessels and accelerate blood circulation, thus strengthening the nutritional supply and metabolism at the lesion site and thereby promoting recovery from disease. Secondly, some components in the crossbow medicinal liquid, such as raw kusnezoff monkshood root, *Gaultheria yunnanensis*, *Periploca forrestii* Schltr., and Chinese alangium root, feature anti-inflammatory, analgesic, and sedative effects [32–34], which can relieve joint pain in patients with KOA. The longer-term effects (WOMAC score, VAS score, Lysholm knee score, and JOA score on 77th day) of crossbow acupuncture might be better than that of acupuncture. But this would require further investigation in a study designed to test superiority. However, we can speculate that as a result of pain relief, the condition of “pain-restricted movement” can be alleviated, and the overall function of the knee joint will gradually improve as daily activities increase. In addition, the mode of transdermal drug delivery might contribute to better penetration and absorption of the effective components in the crossbow medicinal liquid and thus elevate the bioavailability of the drugs, allowing the therapy to play a positive therapeutic role for KOA. The gradual accumulation of drug absorption and release might explain why during follow-up, symptoms further improved.

Studies have previously shown that Miao crossbow needle therapy could reduce the WOMAC score of patients after treatment, reduce the pain and stiffness symptoms of patients with KOA, and improve their joint function [13, 35]. One suggested the clinical effect of crossbow needle therapy may be related to the reduction of serum interleukin-1 and tumor necrosis factor-α levels [35]. While the other study showed that crossbow therapy could increase the hyaluronic acid content in the joint fluid of patients with KOA and reduce the nitric oxide content in the joint fluid, thereby reducing the knee pain and improving joint function [13]. The above studies preliminarily explained the possible mechanism for the clinical effect of Miao crossbow needle therapy on KOA, which could explain the effects observed in the present study. Nevertheless, relevant studies into the mechanisms involved are still lacking, so the possible mechanism of the effect of Miao crossbow needle therapy needs to be verified by subsequent studies.

### Limitations of the study

Since the extraction of knee joint fluid would cause trauma, which would then hinder the following rolling acupuncture treatment on the knee surface using the needle roller, detection of the changes in knee joint fluid of participants was not designed in our protocol. The insufficient objective indicators required to illustrate the clinical efficacy of Miao crossbow needle therapy will be optimized in future research designed to explore the mechanism of crossbow therapy. Another limitation is that no blank control group was set. In addition, the efficacy of crossbow acupuncture in the treatment of KOA was not based on objective measures, but on subjective measures of the patient on a scale. Finally, no cost analysis was performed.

## Conclusions

Both Miao crossbow needle therapy and acupuncture reduced the WOMAC score. The results of the WOMAC, JOA, Lysholm knee score, and VAS scales on the 77th day suggested that the longer-term effect of crossbow acupuncture might be better than that of acupuncture. This therapy can be used as an alternative therapy for KOA.

## Data Availability

The datasets used or analyzed (or both) during the current study are available from the corresponding author on reasonable request.

## Abbreviations

CN: Crossbow needle
AT: Acupuncture
KOA: Knee osteoarthritis
WOMAC: Western Ontario and McMaster Universities Osteoarthritis Index
JOA: Japanese Orthopedic Association
VAS: Visual analog scale
SF-36: MOS 36-item short-form health survey
IWR: Interactive web response
CRF: Case report form
